# Hospital implementation of health information technology and quality of care: are they related?

**DOI:** 10.1186/1472-6947-12-109

**Published:** 2012-09-27

**Authors:** Joseph D Restuccia, Alan B Cohen, Jedediah N Horwitt, Michael Shwartz

**Affiliations:** 1 Health Policy Institute, Boston University School of Management, 53 Bay State Road, Boston, MA, 02215, USA; 2 VA Boston Healthcare System, 150 South Huntington Avenue, Boston, MA, 02130, USA

## Abstract

**Background:**

*Recently, there has been considerable effort to promote the use of health information technology (HIT) in order to improve health care quality. However, relatively little is known about the extent to which HIT implementation is associated with hospital patient care quality*. *We undertook this study to* determine the association of various HITs with: hospital quality improvement (QI) practices and strategies; adherence to process of care measures; risk-adjusted inpatient mortality; patient satisfaction; and assessment of patient care quality by hospital quality managers and front-line clinicians.

**Methods:**

We conducted surveys of quality managers and front-line clinicians (physicians and nurses) in 470 short-term, general hospitals to obtain data on hospitals’ extent of HIT implementation, QI practices and strategies, assessments of quality performance, commitment to quality, and sufficiency of resources for QI. Of the 470 hospitals*, 401 submitted complete data necessary for analysis.* We also developed measures of hospital performance from several publicly data available sources: *Hospital Compare adherence to* process of care measures; Medicare Provider Analysis and Review (MEDPAR) file; and Hospital Consumer Assessment of Healthcare Providers and Systems HCAHPS® survey. We used Poisson regression analysis to examine the association between HIT implementation and QI practices and strategies, and general linear models to examine the relationship between HIT implementation and hospital performance measures.

**Results:**

Controlling for *potential* confounders, we found that hospitals with high levels of HIT implementation engaged in a statistically significant greater number of QI practices and strategies, and had significantly better performance on mortality rates, patient satisfaction measures, and assessments of patient care quality by hospital quality managers; *there was weaker evidence of higher assessments of patient care quality by front-line clinicians.*

**Conclusions:**

Hospital implementation of HIT was positively associated with activities intended to improve patient care quality and with higher performance on *four* of six performance measures.

## Background

Interest in the role of health information technology (HIT) for improving health care quality and patient safety has grown dramatically in recent years, spurred by the Institute of Medicine’s 2001 report, *Crossing the Quality Chasm*, that emphasized “the critical role of information technology in the design of health care systems” to meet six aims of care, i.e., care “that is safe, effective, efficient, timely, equitable and patient-centered” [1]. The report recommended establishing a healthcare information infrastructure that would lead to the elimination of most handwritten clinical data by the end of the decade. Since then, the federal government has established an Office of the National Coordinator for Health Information Technology (ONC) within the Department of Health and Human Services; various private organizations, such as the Institute for Healthcare Improvement (IHI) and the Leapfrog Group, have made HIT adoption a central theme within their quality improvement (QI) campaigns; and numerous healthcare providers have invested substantially in acquiring various HITs. The passage of the Health Information Technology for Economic and Clinical Health Act (HITECH), as part of the American Recovery and Reinvestment Act of 2009, included over $20 billion for HIT, and provided further indication of the growing consensus regarding the potential salutary effect of HIT [2].

HITs intended to improve patient care quality and safety encompass an array of technologies, most notably electronic medical records (EMRs), computerized provider order entry (CPOE) systems, medication management systems (MMS), and picture archival and communications systems (PACS), all designed to improve the accuracy, accessibility, and timeliness of storage and transmission of patients’ medical information. Two other technologies—bar coding and radio frequency identification (RFID) systems—are used to track the location and disposition of pharmaceuticals, medical equipment, surgical supplies, and patients to help ensure, for example, that medications are administered safely and correctly.

Despite the growing interest in information technology, relatively little is known about the extent to which HIT implementation is associated with hospital patient care quality. A systematic review of 257 studies of the impact of HIT found few studies that have shown an impact on quality [3]. Of these, the most important positive impact was on adherence to guideline-based or protocol-based care through use of decision support systems providing computerized reminders for preventive care, such as vaccinations and blood tests. Moreover, most such studies involved a single technology and a single site, often in academic medical centers, thus limiting their generalizability to broad-based use of HIT or to other types of healthcare provider organizations. We found *six* other articles investigating the relationship between HIT and quality of care in multiple sites that have been published since 2006. Amarasingham et al. [4] reported a study involving a sample of 41 Texas hospitals that found that the extent of automation of clinical information processes was associated at statistically significant levels with lower inpatient mortality and fewer patient complications. In a study involving 2,707 hospitals, Parente and McCullough [5] investigated the association between three HITs (EMRs, nurse charts, and PACS) and three patient safety indicators (infection due to medical care, postoperative haemorrhage or hematoma, and pulmonary embolism or deep vein thrombosis). The only statistically significant association found was between EMRs and reduced infections due to medical care. McCullough et al. [6] found that, among 3,401 hospitals classified into those with both an EMR and CPOE and those without either of these HITs, the former showed small but statistically significant improvement between 2004 and 2007 for two of six process measures of quality (pneumococcal vaccine administration and use of the most appropriate antibiotic for pneumonia). Himmelstein et al. [7] developed a “computerization score” for 4,000 hospitals and found that it was weakly related to process measures for acute myocardial infarction but not for heart failure, pneumonia, or a composite of the three conditions. Mollon et al. [8] conducted a systematic review of studies to evaluate the effect of prescribing decision support systems on patient outcomes. Only five of the 41 studies that met their inclusion criteria, primarily that the study design was a randomized controlled trial, reported improvements in patient outcomes. *Encinosa and Bae*[9]*studied the relationship between hospital EMR use and the outcomes and cost of hospital care in a sample of 2,619 institutions. They found that EMRs had no impact on the rate of patient safety events, although having an EMR assisted in responding to an event, reducing deaths, readmissions, and expenditures.*

In this paper, we report findings from a study involving *401* U.S. hospitals that examined the relationship between the level of hospital HIT implementation and use of QI practices and strategies as well as with performance on five sets of quality of care measures: 1) adherence to the Hospital Compare process of care measures for acute myocardial infarction (AMI), heart failure (HF) and pneumonia; 2) risk-adjusted inpatient mortality; 3) patient satisfaction, as derived from the Hospital Consumer Assessment of Healthcare Providers and Systems (HCAHPS®) survey; 4) hospital quality managers’ assessments of patient care quality; and 5) front-line clinicians’ assessments of patient care quality.

## Methods

### Sample design

We designed and conducted a survey in 2006 of all 4,237 short-term, non-federal, general service hospitals in the United States that had at least 25 beds, according to the 2004 AHA Annual Survey of Hospitals. Pediatric, psychiatric, rehabilitative, orthopedic, and chronic disease hospitals were excluded from the sample.

### Survey content

We developed and administered two surveys, the *Quality Improvement Activities Survey* (*QAS*) and the *Clinicians’ Perceptions of Quality Survey* (*CPS*). The *QAS* instrument was intended for completion by the hospital’s chief quality officer (CQO) or designated lead quality manager, and was designed to gather information about the nature and extent of QI activities undertaken and their impact on patient care quality. The *CPS* was intended for completion by physicians and nurses to elicit their assessment of patient care quality at their hospital. The questionnaires contained mostly new and unique items, but also included some questions adapted from established surveys, such as the National Survey of Efforts to Improve Quality [10] and the Leapfrog Group’s Hospital Quality and Safety Survey [11], as well as questions regarding several QI activities endorsed by the Institute for Healthcare Improvement in its 100,000 Lives Campaign. Some questions also were adapted from the first-wave survey instrument developed by members of our team, in collaboration with colleagues from Boston University and the VA Boston Healthcare System, for an evaluation of the Robert Wood Johnson Foundation’s *Pursuing Perfection Program*[12]. The final versions of the *QAS* and *CPS* were derived based on pilot testing in a small sample of hospital CQOs and physicians and nurses, respectively, and on feedback from experienced health services researchers with expertise in survey research. The final version of the *QAS* contained 173 questions and took approximately 45 minutes to complete while the *CPS* contained 74 questions and required about 20 minutes to complete. The study design, instruments, and informed consent procedures were approved by the Institutional Review Boards of Boston University and the Health Research & Educational Trust (HRET).

Hospital quality managers were asked to indicate the extent to which eight HITs had been implemented in their hospitals, using a six-point scale with the following response categories: “not under consideration;” “under active discussion but not yet budgeted;” “budgeted but not yet in place;” “in testing;” “implemented in one or more units;” and “implemented hospital-wide.” The HITs included: 1) inpatient Electronic Medical Record (EMR) System, 2) outpatient EMR System, 3) inpatient Computerized Provider Order Entry (CPOE) System, 4) outpatient CPOE System, 5) Medication Management System (MMS), 6) Picture Archival and Communications System (PACS), 7) bar coding, and 8) Radio Frequency Identification (RFID) technology. In addition to assessing the extent of HIT implementation in hospitals, the *QAS* included questions on the extent of implementation of specific quality practices and clinical strategies used throughout the hospital*, asked on a 5-point scale* anchored by “not used at all” and “used hospital-wide,” eight of which could be expected to be facilitated by HIT [Table 1]. Two questions asked of clinicians in the *CPS*, on a five-point scale anchored by “strongly disagree” and “strongly agree,” were the extent to which “the hospital is committed to delivering the highest quality patient care” and whether “the hospital provides sufficient resources and support for improving patient care.” (We shall subsequently refer to these as the “commitment question” and the “resources question.”) A question common to both surveys asked respondents how they would rate patient care today at their hospital compared to what they think it should be on a five-point scale ranging from “well below expectations” to “well above expectations.”

**Table 1:** Hospital Quality Practices and Strategies Potentially Facilitated by HIT Implementation and Use

1	Progress toward achieving hospital-wide quality goals is tracked and communicated to clinical staff
2	Quality improvement project results are regularly communicated to clinical staff
3	The hospital regularly communicates achievement of hospital-wide quality goals to the general public
4	Patient care processes are standardized, where and when appropriate
5	Evidence-based practice guidelines/clinical pathways
6	Chronic disease registries
7	Standing orders
8	Medication reconciliation

In the analyses in this paper, we recoded responses to questions asking extent of agreement so that “agree” or “strongly agree” were coded as 1 and the other three response categories were coded as 0. Similarly, we recoded responses to questions about implementation so that “used hospital-wide” and “used widely” were coded as 1 and the other three categories were coded as 0. The *CPS* was administered to a random sample of physicians and nurses in each hospital based on hospital bed size, ranging from 6 in small hospitals to 12 in large hospitals.

General findings from the *QAS* and a detailed description of the survey’s complex methodology are reported elsewhere [13].

### Final sample

The sample contained 470 hospitals *that submitted surveys,* representing 11 percent of the 2004 population from which they were drawn. Eight survey responses failed to provide complete answers to questions regarding HIT implementation*. In addition, we included in our analysis only hospitals that had a response from the CQO and responses to the CPS from at least three front-line clinicians. This reduced the final sample size to 401 hospitals.* The length and complexity of the questionnaires contributed to the lower-than-desired response rate. However, as reported in Cohen et al. [13], the sample of 470 hospitals was similar to the population of hospitals (2005 AHA Annual Survey, n = 4,222) along a number of dimensions *including Census region (Midwest, Northeast, South, and West), network affiliation, system affiliation, Medicare disproportionate share hospital status, and location in a metropolitan or non-metropolitan county* (*as is the current study’s sample of 401 hospitals of these 470 hospitals)*. The main differences between the population and the sample in the current study were the higher percentages in our sample of large hospitals (*19.5% with over 400 beds* vs. 9.7% in the population) and teaching hospitals (*15.4% with membership in the Council of Teaching Hospitals* vs. 6.5% in the population) and the smaller percentage of for-profit hospitals (*3.6%* vs. 15.7% in the population).

In addition, to understand the extent to which hospitals responding to the survey may be different in terms of their commitment to QI, we compared sample hospitals to the population of hospitals in terms of their performance on the 15 Hospital Compare process measures for acute myocardial infarction (AMI), congestive heart failure (CHF) and pneumonia (described in more detail in the next section). Using the approach described in the next section, we calculated composite measures of both overall performance on the process measures and condition-specific performance. When the overall composite measure was divided into deciles, the average hospital in the population fell into the fifth decile, while the average hospital in the sample was one decile higher in terms of quality. Similar results were obtained when analyses were conducted separately for each of the three conditions. Thus, while the differences were not large, the better performance on Hospital Compare measures among hospitals responding to the survey suggested that they may be in the vanguard of QI efforts (i.e., more likely to have embraced QI aims and to have engaged more extensively in QI activities) than non-participating hospitals.

### Quality of care measures - hospital compare

#### Process of care measures

We developed a composite measure of hospital processes of care based on the Hospital Compare data for three conditions: acute myocardial infarction, heart failure, and pneumonia. We used data available from the Centers for Medicare and Medicaid Services (CMS) Hospital Compare website for calendar year 2005 on adherence to the evidence-based processes of care from hospitals that had at least 100 patients eligible for the sum of the following 15 process measures for the three conditions: AMI (*6 measures*): aspirin at arrival; aspirin prescribed at discharge; ACE inhibitor or angiotensin receptor blocker (ARB) for left ventricular systolic dysfunction (LVSD); beta blocker prescribed at discharge; beta blocker at arrival; and adult smoking cessation advice/counsel; HF (*4 measures)*: left ventricular function assessment; angiotensin-converting enzyme (ACE) inhibitor or ARB for LVSD; discharge instructions; and adult smoking cessation advice/counselling; Pneumonia *(5 measures)*: oxygenation assessment; pneumococcal vaccination status assessment; initial antibiotic received within 4 hours of hospital arrival; blood culture performed in emergency department before first antibiotic received in hospital; and adult smoking cessation advice/counselling.

To calculate a composite measure across all 15 process measures, we used the approach recommended by CMS in its Premier demonstration pay-for-performance program for aggregating across measures within condition: sum the numerators, sum the denominators, and then calculate the ratio of summed numerators to summed denominators [14]. This is equivalent to calculating a weighted average of the proportion eligible for each intervention that receives the intervention, where the weight applied to each proportion is the ratio of the number eligible for the specific intervention to the sum of the numbers of eligibles for all interventions. These weights are called opportunity-based weights. We calculated the composite measure for all hospitals where the sum of the numbers of those eligible for each of the interventions was greater than 100.

### Inpatient mortality rates

We applied the 3 M™ Health Information Systems’ All Patient Refined Diagnosis Related Groups (APR-DRGs) software to the CMS Medicare Provider Analysis and Review (MEDPAR) File to measure patient severity. The APR-DRG software adds four subclasses to each DRG based on mortality risk. Using a reference population of 4.5 million Medicare patients from approximately 1,000 hospitals (including the *401* hospitals in this study) [15], we calculated the risk of in-hospital mortality for each subclass in each DRG and then assigned each patient in our sample of *401* hospitals an expected mortality risk based on their DRG subclass. The expected number of deaths in each hospital was calculated by summing the expected mortality risks of all patients in that hospital. We then calculated the ratio of observed deaths to expected deaths (O/E) considering only those patients who had one of the conditions that comprise the AHRQ *Mortality* Inpatient Quality Indicators *(*https://www.qualityindicators.ahrq.gov*),* as these conditions have been judged to be ones in which in-hospital mortality is sensitive to the quality of patient care provided.

### Patient satisfaction

We downloaded from the CMS website HCAHPS® data for sampled hospitals. We considered two questions from the survey:

1) How do you rate the hospital overall?

2) Would you recommend the hospital to friends and family?

For the first question, we focused on the percentage of respondents who gave the hospital a rating of 9 or 10 (the two highest ratings). For the second question, we focused on the percentage of respondents who said they would definitely recommend the hospital. We used the average response for the two questions as the measure of patient satisfaction.

### Hospital quality Managers' assessments of patient care quality

This measure consisted of the response by the hospital quality manager to the question, “How would you rate patient care today at your hospital compared to what you think it should be?”

### Front-line Clinicians' assessments of patient care quality

This measure consisted of the average of responses by front-line clinicians (physicians and nurses) to the question above that was asked of quality managers. We first calculated the mean assessment in each hospital and then used these means in the analysis. We preferred this approach to one that uses the individual front-line clinician response as the unit of analysis because it weights each hospital equally, as opposed to giving more weight to larger hospitals with greater numbers of front-line clinician responses. We have shown that in this sample it is reasonable to aggregate individual responses to the hospital level [15].

### Statistical analysis

For each performance measure, we *used one-way ANOVA to examine the difference in the average of the performance measure by the following* HIT categories: 0 or 1 (low), 2 to 4 (medium), and 5 or more (high). To identify pairs of means that differed across HIT category, we used Tukey’s HSD (honestly significant difference) test.

When performing the statistical analyses for mortality, which is in the form of an O/E ratio, we took the log of the ratio before performing the analysis. When examining the performance measure “assessment of quality by front-line clinicians,” we first calculated the mean assessment in each hospital and then used these means in the analysis.

To investigate the relationship between extent of HIT implementation and the performance measures, we used a General Linear Model (GLM) with *the following independent variables: the HIT categories defined above, hospital* structural characteristics, and the mean clinician response by hospital for the commitment and resource questions. We included the following four structural characteristics: bed-size category *(25–99 beds, 100–399 beds, >400 beds)*, ownership type *(government, not-for-profit, for-profit)*, urban/rural *location (metropolitan county or non-metropolitan county)*, and teaching status (accredited member *or non-member* of the Council of Teaching Hospitals and Health Systems). In addition, we included as independent variables clinicians’ responses to the commitment question and to the resources question, *both of which might seriously confound the relationship between HIT and the performance measures.* The assumption underlying inclusion of these two variables is that commitment and resources are the drivers of quality; HIT is one of the important means by which commitment and resources are translated into improved performance. This leads to our specific hypothesis: among hospitals with the same level of commitment and resources, those that have more completely implemented HIT will have higher levels of performance. However, there is an alternative hypothesis one might reasonably make: survey responders believe commitment and resources are higher when HIT is more fully implemented. That is, assessed levels of commitment and resources reflect the extent of HIT implementation. Under this assumption, commitment and resources should not be included as covariates in the model*. We think the first hypothesis is the most likely and, hence, for our main analyses, we include commitment and resources in the model. Since these variables are positively correlated with extent of HIT implementation, inclusion of the variables decreases the chance of finding a statistically significant relationship between HIT and performance. When extent of HIT implementation was not statistically significant, we reran the model without these variables.*

To examine the relationship between extent of QI practices and strategies used in the hospital and extent of HIT implementation, we ran a Poisson regression model with the number of practices and strategies as the dependent variable and the same independent variables as above.

We interpreted *p* values of less than 0.05 as indicating statistically significant differences. Survey data were analyzed using SPSS version 16.0.

## Results

Table 2 shows the unadjusted means for each of the performance measures. For all of the performance measures, there was a statistically significant difference by HIT category and between the low HIT category and the high HIT category. For some measures, there were also statistically significant differences between the low category and the medium category, or between the medium category and the high category.

**Table 2:** Relationships between HIT Implementation and Mean Number of QI Strategies and Practices, and between HIT Implementation and Mean of the Hospital Performance Measures (numbers of hospitals in parentheses)

**Measure**	**HIT Category**			**P-value**
	**Low**	**Medium**	**High**	
QI Strategies and Practices	2.44 (79)	3.63 (264)	4.20 (61)	0.000 †^§^
Composite HQA Process of Care	81.0 (51)	82.6 (241)	85.2 (61)	0.009 †^‡^
Observed to Expected Mortality Rate*	1.29 (70)	1.06 (263)	1.07 (61)	0.000†^§^
HCAHPS Patient Satisfaction	60.5 (31)	66.0 (187)	67.9 (48)	0.000†^§^
Quality Manager Assessment of Patient Care Quality	3.11 (81)	3.19 (266)	3.56 (62)	0.001†^‡^
Front-line Clinicians’ Assessment of Patient Care Quality	3.22 (82)	3.31 (269)	3.40 (62)	0.032†

†Significant difference between Low and High.

^‡^Significant difference between Medium and High.

^§^Significant difference between Low and Medium.

*: ln(O/E) was used when conducting the statistical tests.

Hospitals in the high HIT implementation category used an average of 4.20 practices and strategies while those in the medium category used 3.63 and those in the low category used 2.44 (p < 0.000 for differences in unadjusted means). As seen in Table 3, which contains the parameter estimates of the multivariable models for each of the six performance measures, after controlling for covariates, we still found that hospitals with high levels of HIT implementation, engaged in significantly greater numbers of HIT-related QI practices and strategies (p = 0.003 for differences in adjusted means).

**Table 3:** Multivariable Model Parameter Estimates

Covariate*	***QI Strategies and Practices***			***Composite HQA Process of Care***			***Observed to Expected Mortality Rate***		
	**Beta**	**95% CI**	**P**	**Beta**	**95% CI**	**P**	**Beta**	**95% CI**	**P**
bed-size category			0.285			0.022			0.972
25-99 beds	−0.177	−0.398, 0.045	0.119	−0.04	−0.07, -0.010	0.009	0.007	−0.123, 0.137	0.916
100-399 beds	−0.057	−0.205, 0.091	0.453	−0.011	−0.032, 0.010	0.308	−0.004	−0.103, 0.095	0.940
ownership type			0.438			0.001			0.626
government	−0.159	−0.404, 0.086	0.204	−0.003	−0.046, 0.039	0.881	−0.017	−0.205, 0.170	0.856
not-for-profit	−0.122	−0.334, 0.090	0.260	0.034	−0.006, 0.073	0.093	−0.053	−0.229, 0.124	0.557
urban	0.079	−0.078, 0.236	0.323	−0.009	−0.029, 0.010	0.353	−0.098	−0.181, -0.014	0.022
non-teaching	0.046	−0.113, 0.205	0.569	0.006	−0.017, 0.029	0.607	0.050	−0.056, 0.156	0.350
HIT category			**0.003**			**0.257**			**0.005**
low	−0.378	−0.610, -0.146	0.001	−0.021	−0.049, 0.008	0.157	0.065	−0.061, 0.191	0.312
medium	−0.084	−0.236, 0.068	0.277	−0.016	−0.037, 0.004	0.122	−0.076	−0.172, 0.020	0.121
commitment	0.123	−0.091, 0.336	0.261	0.03	0.002, 0.058	0.037	−0.029	−0.155, 0.096	0.646
resources	0.090	−0.085, 0.265	0.312	0.007	−0.015, 0.029	0.545	−0.010	−0.109, 0.089	0.842
R^2^	0.109			0.136			0.072		
	***HCAHPS Patient Satisfaction***			***Quality Manager Assessment of Patient Care Quality***			***Front-line Clinicians’ Assessment of Patient Care Quality***		
Covariate*	**Beta**	**95% CI**	**P**	**Beta**	**95% CI**	**P**	**Beta**	**95% CI**	**P**
bed-size category			0.002			0.153			0.006
25-99 beds	2.390	−1.493, 6.273	0.227	0.119	−0.186, 0.424	0.443	0.112	0.004, 0.220	0.042
100-399 beds	−2.256	−4.977, 0.466	0.104	−0.082	−0.316, 0.153	0.493	−0.011	−0.094, 0.072	0.797
ownership type			0.001			0.613			0.094
government	8.952	3.467, 14.437	0.001	0.207	−0.221, 0.634	0.343	0.168	0.015, 0.320	0.031
not-for-profit	9.373	4.400, 14.347	0.000	0.199	−0.203, 0.601	0.331	0.130	−0.013, 0.274	0.075
urban	−0.149	−2.569, 2.271	0.903	0.056	−0.137, 0.249	0.570	−0.021	−0.089, 0.048	0.557
non-teaching	−1.220	−4.030, 1.590	0.393	−0.030	−0.280, 0.220	0.813	−0.097	−0.186, -0.008	0.032
HIT category			**0.000**			**0.006**			**0.392**
low	−7.208	−11.047, -3.680	0.000	−0.439	−0.730, -0.147	0.003	−0.067	−0.170, 0.036	0.202
medium	−1.177	−3.776, 1.421	0.370	−0.342	−0.567, -0.117	0.003	−0.022	−0.102, 0.058	0.586
commitment	2.686	−0.889, 6.261	0.140	0.337	0.050, 0.623	0.021	0.370	0.268, 0.473	0.000
resources	2.814	0.029, 5.599	0.048	0.022	−0.209, 0.253	0.851	0.354	0.271, 0.436	0.000
R^2^	0.228			0.078			0.579+		

* The reference category is excluded for each covariate, e.g. estimates for non-teaching hospitals are relative to teaching hospitals.

+ High R^2^ is due to the strong relationship between the commitment question and the quality performance measure question.

Risk-adjusted inpatient mortality was higher for hospitals with low HIT implementation compared to those with medium or high HIT implementation, with the O/E ratio for the former being 1.29, compared to 1.06 and 1.07 for the latter two, respectively (p < 0.000 unadjusted; p = 0.005 adjusted).

The HCAHPS®-based measure of patient satisfaction showed a similar finding, with low HIT implementation hospitals having a 60.5% average satisfaction score and medium and high HIT implementation hospitals having average scores of 66.0% and 67.9%, respectively (p < 0.000 unadjusted; p < 0.000 adjusted).

Quality managers’ assessments of patient care quality (i.e., responses to the question of how they would rate patient care today at their hospital compared to what they think it should be) were higher for hospitals that had higher levels of HIT implementation. The average scores, on a five-point scale, for high, medium, and low HIT implementation hospitals were 3.56, 3.19, and 3.11, respectively (p = 0.001 unadjusted; p = 0.006 adjusted). For front-line clinicians’ assessments of quality, differences between unadjusted means (with average scores of 3.40, 3.31, and 3.22, respectively) were significant (p = 0.032). After covariate adjustment, they were not significant (p = 0.392). However, if the commitment and resource variables were not included in the multivariable adjustment model, the difference in means by HIT category was statistically significant (p < 0.000).

The percent adherence to the composite Hospital Compare process of care measure increased with greater HIT implementation, with low HIT implementation hospitals at 81.0% adherence, medium HIT implementation hospitals at 82.6% adherence, and high HIT implementation hospitals at 85.23% adherence (p = .009). However, the differences were not statistically significant in the multivariable model either with or without inclusion of the commitment and resource as covariates.

## Discussion

We found a statistically significant association between the extent of HIT implementation and individual hospital quality practices and strategies that could be facilitated by HIT, plus a statistically significant association between HIT implementation and hospital performance on four of five measures of quality (though in one case, front-line clinicians’ assessment of quality, the results were statistically significant only when the commitment and resources questions were not included in the model).

It is likely that HITs are enablers of quality practices and clinical QI strategies through enhanced communication, documentation, information transfer, performance monitoring, and error prevention, thus, leading to improved quality performance.

A limitation of the study is that of the performance measures associated with HIT implementation, one was based on the quality manager survey in which respondents were asked about both HIT implementation and patient care quality. This creates a common methods bias and makes it difficult to draw conclusions about causality. It is possible that respondents may have believed that patient care quality was better in their hospitals simply because their hospitals had implemented quality-enhancing HITs. However, the two publicly-available measures that showed a relationship to HIT implementation, mortality rate and patient satisfaction, are not subject to common methods bias. It is unlikely that knowledge of the hospital’s performance on these measures influenced survey respondents to indicate a particular level of HIT implementation.

Another limitation is the survey response rate of 11 percent for the two survey-assessed measures of patient care quality. We cannot rule out the possibility that unmeasured, complex motivational factors may have contributed to the selective response by hospitals to participate in the surveys. As described in the Methods section, teaching hospitals were overrepresented in the sample and for-profit and non-metropolitan hospitals were underrepresented. In addition, sample hospitals, on average, performed better on Hospital Compare measures [13]. Although the differences were not large, the sample hospitals’ higher performance levels on these measures suggested that they may have been in the vanguard of QI efforts than non-participating hospitals. Thus, our findings are not necessarily representative of all short-term, non-federal, general service hospitals with 25 or more beds. However, given that the study includes over 400 hospitals, study findings nevertheless provide important information on the relationship between HIT implementation and quality of care. Furthermore, because the observed levels of HIT implementation and performance in sample hospitals still fell well below targets set by the Institute of Medicine and other QI proponents, our results suggest that there is substantial room for improvement even in hospitals that appear to be more advanced than many.

Further research is needed to determine the generalizability of the relationship between HIT implementation and quality of care, and to ascertain the particular features of health information systems that lead to effective QI activities and quality performance. However, it is clear that, for the 401 hospitals in our study, those with higher levels of HIT implementation were more likely to engage in practices and strategies intended to improve the quality of patient care and also exhibited better performance on important measures reflecting different dimensions of quality: a clinical outcomes measure (risk-adjusted mortality); a publicly-available measure of patient satisfaction (HCAHPS®); assessment of patient care quality by hospital quality managers; *and, though the evidence was weaker, assessment of quality by front-line clinicians.*

## Conclusions

For many years, the federal government and private organizations, such as the Institute of Medicine and the Leapfrog Group, have encouraged increased investment in information technologies, most notably EMR and CPOE systems, to improve patient care quality and safety. Numerous barriers to HIT implementation have been posited, among them high cost, technological complexity, decreased physician productivity, and uncertain return on investment [16]. Clearly, these barriers must be overcome if nationwide levels of HIT implementation are to increase substantially, especially in small, non-teaching, non-metropolitan hospitals, which lag behind their larger, academic, urban counterparts [17]. Our study provides empirical evidence that such efforts may be warranted.

## Abbreviations

AMI: Acute Myocardial Infarction; AHRQ: Agency for Healthcare Research and Quality; APR-DRGs: All Patient Refined Diagnosis Related Groups; AHA: American Hospital Association; ARB: Angiotensin Receptor Blocker; ACE: Angiotensin-Converting Enzyme; CMS: Centers for Medicare and Medicaid Services; CQO: Chief Quality Officer; *CPS*: *Clinicians’ Perceptions of Quality Survey*; CPOE: Computerized Provider Order Entry; EMRs: Electronic Medical Records; GLM: General Linear Model; HITs: Health Information Technologies; HITECH: Health Information Technology for Economic and Clinical Health Act; HRET: Health Research & Educational Trust; HF: Heart Failure; HCAHPS: Hospital Consumer Assessment of Healthcare Providers and Systems; IHI: Institute for Healthcare Improvement; IOM: Institute of Medicine; LVSD: Left Ventricular Systolic Dysfunction; MEDPAR: Medicare Provider Analysis and Review; MMS: Medication Management Systems; ONC: Office of the National Coordinator for Health Information Technology; PACS: Picture Archival and Communications Systems; QI: Quality Improvement; *QAS*: *Quality Improvement Activities Survey*; RFID: Radio Frequency Identification; HSD: Tukey’s Honestly Significant Difference.

## Competing interests

The authors declare that they have no competing interests.

## Authors’ contributions

JR conceived the study. JR, AC, and MS contributed to the research design. JR and AC obtained funding. MS, JH, and JR were involved in the data analysis. All authors were involved in the interpretation of data and have read and given final approval of paper.

## Pre-publication history

The pre-publication history for this paper can be accessed here:

http://www.biomedcentral.com/1472-6947/12/109/prepub
